# In vivo evaluation of additively manufactured multi-layered scaffold for the repair of large osteochondral defects

**DOI:** 10.1007/s42242-021-00177-w

**Published:** 2022-03-16

**Authors:** Maryam Tamaddon, Gordon Blunn, Rongwei Tan, Pan Yang, Xiaodan Sun, Shen-Mao Chen, Jiajun Luo, Ziyu Liu, Ling Wang, Dichen Li, Ricardo Donate, Mario Monzón, Chaozong Liu

**Affiliations:** 1 Institute of Orthopaedic and Musculoskeletal Science, Royal National Orthopaedic Hospital, University College London, Stanmore, HA7 4LP UK; 2 School of Pharmacy and Biomedical Sciences, University of Portsmouth, Portsmouth, PO1 2DT UK; 3 Guangdong Engineering Research Center of Implantable Medical Polymer, Shenzhen Lando Biomaterials Co., Ltd., Shenzhen, 518107 China; 4 School of Materials Science and Engineering, Tsinghua University, Beijing, 100084 China; 5 State Key Laboratory for Manufacturing System Engineering, School of Mechanical Engineering, Xi’an Jiaotong University, Xi’an, 710054 China; 6 Departamento de Ingeniería Mecánica, Grupo de Investigación en Fabricación Integrada y Avanzada, Universidad de Las Palmas de Gran Canaria, Campus Universitario de Tafira s/n, 35017 Las Palmas, Spain

**Keywords:** Osteochondral scaffold, Large animal, Additive manufacturing, Porous titanium

## Abstract

The repair of osteochondral defects is one of the major clinical challenges in orthopaedics. Well-established osteochondral tissue engineering methods have shown promising results for the early treatment of small defects. However, less success has been achieved for the regeneration of large defects, which is mainly due to the mechanical environment of the joint and the heterogeneous nature of the tissue. In this study, we developed a multi-layered osteochondral scaffold to match the heterogeneous nature of osteochondral tissue by harnessing additive manufacturing technologies and combining the established art laser sintering and material extrusion techniques. The developed scaffold is based on a titanium and polylactic acid matrix-reinforced collagen “sandwich” composite system. The microstructure and mechanical properties of the scaffold were examined, and its safety and efficacy in the repair of large osteochondral defects were tested in an ovine condyle model. The 12-week in vivo evaluation period revealed extensive and significantly higher bone in-growth in the multi-layered scaffold compared with the collagen–HAp scaffold, and the achieved stable mechanical fixation provided strong support to the healing of the overlying cartilage, as demonstrated by hyaline-like cartilage formation. The histological examination showed that the regenerated cartilage in the multi-layer scaffold group was superior to that formed in the control group. Chondrogenic genes such as aggrecan and collagen-II were upregulated in the scaffold and were higher than those in the control group. The findings showed the safety and efficacy of the cell-free “translation-ready” osteochondral scaffold, which has the potential to be used in a one-step surgical procedure for the treatment of large osteochondral defects.

**Graphic abstract:**

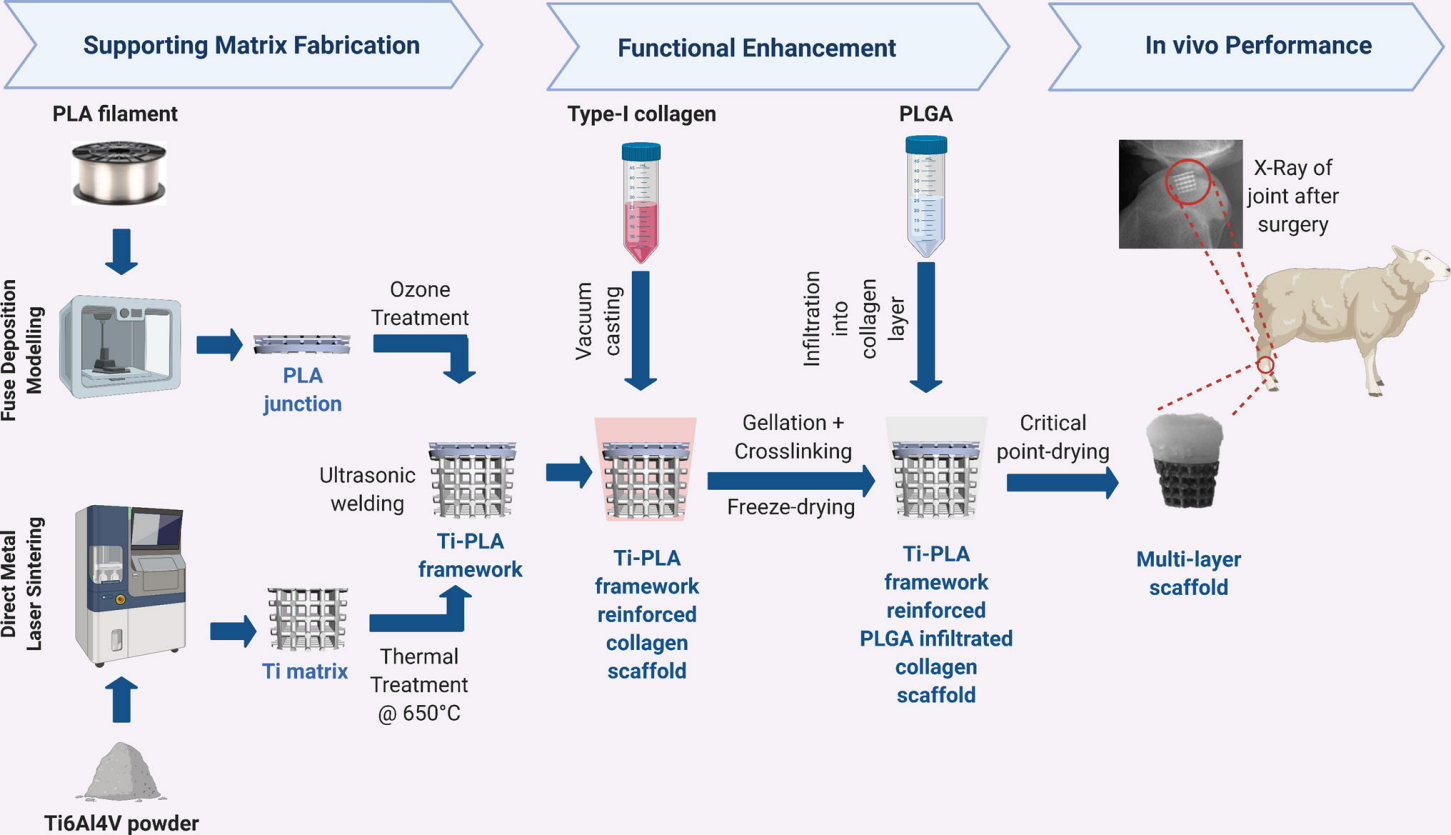

**Supplementary Information:**

The online version contains supplementary material available at 10.1007/s42242-021-00177-w.

## Introduction

Osteochondral defects typically arise from repetitive trauma within the joint, possibly altering the architecture or composition of the bone. Traumatic osteochondral defects affect both the cartilage and the bone. If untreated, these defects will lead to the development of osteoarthritis (OA), where a joint replacement is applied as end-stage treatment. In 2019, over 200,000 joint replacements were performed in England and Wales, 93% of which were predominantly for OA [1].

There has been a wealth of proposed solutions for osteochondral defects. For example, treatments using tissue engineering methods were established, which have demonstrated promising results provided that they are used to treat small osteochondral defects. In the tissue engineering approach, 3D scaffolds have enabled the uniform delivery of cells and act as a barrier to the invasion of the graft by fibroblasts, which may otherwise induce fibrous repair [2, 3]. Although a lot of research has been carried out on developing osteochondral scaffolds, neither any of the developed products seem to promote satisfactory durable regeneration of cartilage, nor they are suitable for the treatment of large defects [4]. Effective treatment is needed that can prevent or delay the progression of OA and improve the success of healthcare.

The main challenge in developing a “clinically satisfactory” osteochondral scaffold is to recapitulate the graded biomechanics and functionality of the osteochondral tissue unit, which is a bio-composite comprised of the articular cartilage, calcified cartilage, and subchondral bone [5], each with different mechanical properties, morphology, physiology and potential to heal. This requires a multiphasic scaffold taking into account the natural graded functionality of native osteochondral tissue [6, 7] and providing simultaneous cues to the cells that are specific to the regeneration of bone and cartilage. These types of scaffolds should include two or more different materials, composites, bioactive molecules or architectures to induce a significant depth-dependent variation in the relevant properties [7]. Studies using conventional osteochondral scaffolds for the repair and regeneration of osteochondral defects have indicated the formation of fibrocartilage rather than hyaline cartilage [8]. Fibrocartilage is rich in type I collagen and has poor durability compared to the hyaline cartilage [9]. In addition, many of the clinical studies have demonstrated that the subchondral bone often fails to regenerate, and shows a distinguishable boundary of the bony pit, subchondral oedema, sclerosis and subchondral cysts [10–14]. Failure to provide stable biomechanical support for the overlying cartilage results in the newly formed tissue collapsing into the defect, which compromises the proper healing of the cartilage.

An ideal osteochondral scaffold should provide a suitable microenvironment for native cells to grow and promote tissue regeneration [15] while honouring the multi-layered structure of native tissue. To this end, this study developed a multi-layer scaffold taking into account the microstructure and mechanical properties conducive to osteochondral tissue regeneration. Additive manufacturing technologies have provided the pharmaceutical and medical industry with great opportunities to offer more rapid and personalised care to their patients. Despite the high volume of related research, these technologies have not yet been adopted into routine clinical care [16]. A review conducted in 2018 to explore biofabrication in the clinic found a clear shift from the early stages of “idea” and “development” towards the “long-term study” phase in this field [16]. The ability of additive manufacturing to produce complex anatomical geometries makes it an excellent technology to fabricate intricate tissue engineering scaffolds, such as the ones intended for the regeneration of osteochondral tissues. Furthermore, additive manufacturing techniques have the clear advantage of achieving multi-modality features of the osteochondral tissue unit that are required for osteochondral scaffolds [17]. This allows for the processing of a range of biomaterials, including metals [18], into structures with tuneable mechanical properties that could be beneficial in fabricating a biomechanically favourable scaffold for osteochondral tissue regeneration.

With the aim of moving closer from the “discovery” stage to the “translation” phase [19], and to address the complexity of natural osteochondral tissue, we developed an osteochondral scaffold composed of hard and soft materials by integrating different additive manufacturing techniques. The proposed scaffold system is comprised of porous titanium (Ti) layer as the bone component and a polylactic-co-glycolic acid (PLGA) infiltrated collagen layer as the cartilage component, joined together by a porous polylactic acid (PLA) junction layer. All of the materials used for the scaffold fabrication, including Ti, PLA, PLGA and collagen, have been used extensively in clinical settings [2, 20–22]. Regarding safety and efficacy, we have selected established 3D printing methods and biomaterials with a history of safe use in osteochondral scaffold fabrication. The constructed osteochondral scaffold was characterised in terms of microstructure, mechanical properties and biocompatibility, and evaluated using a sheep condyle model to establish its safety and efficacy for the repair of large osteochondral defects.

## Materials and methods

### Osteochondral scaffold design

**Fig. 1:**
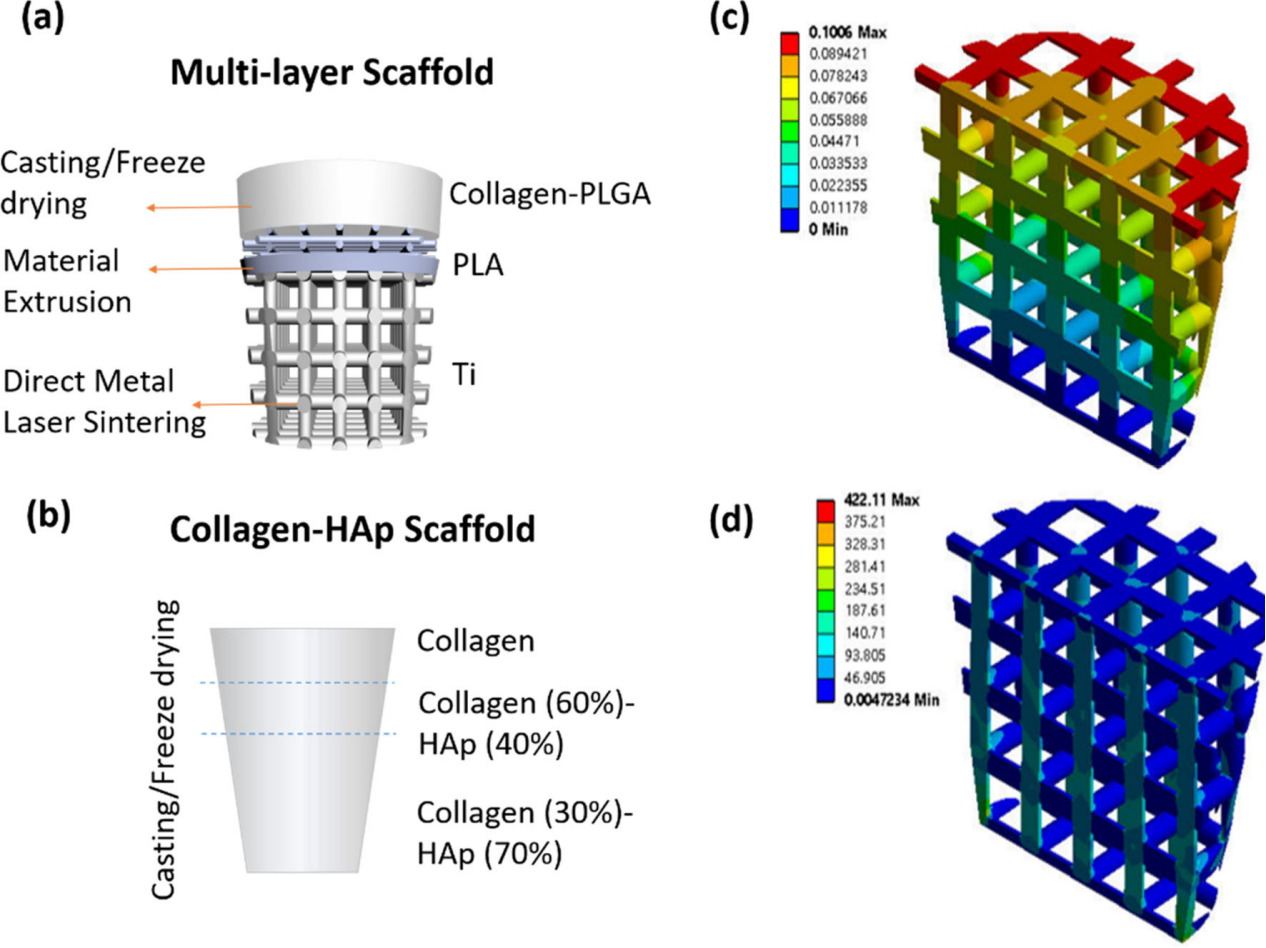
Schematic of **a** the designed multi-layer scaffold, and **b** the collagen–HAp scaffold used in the in vivo study. The finite element analysis of Ti matrix showed that **c** the most of deformation occurred at the top layer of lattice around the edge of the scaffold, and that **d** no equivalent stress concentrations occurred throughout the scaffold

In order to address the complexity of the natural osteochondral tissue, the osteochondral scaffold in this paper is designed as a multi-layered, multi-material structure (multi-layer scaffold) based on a Ti alloy, PLA and collagen–PLGA composite system. Figure 1a shows a schematic design of the trilayered osteochondral scaffold, which has a Ti matrix as the bone component and a PLGA reinforced collagen layer as the cartilage component for cartilage repair. The dense junction layer of PLA acts as “calcified cartilage” to join the cartilage section with the bone section, forming a graded structure with respect to mechanical properties, structure and composition.

The lower layer corresponding to bone tissue is designed as a porous matrix to allow for bone ingrowth and vascularisation. Pore sizes of 300–800 µm are considered beneficial for bone ingrowth [23]; therefore, the Ti matrix was designed with a strut diameter of 0.5 mm and a pore size of 0.75 mm. As the main component of the scaffold to provide mechanical support for the rest of the layers, the stability of the Ti layer was explored in the initial stages of implantation.

Finite element analysis was used to study the stress and strain distribution within the osteochondral scaffold. The stress level of 5 MPa was applied to the surface of Ti, assuming fixation at the bottom surface and no deformation in the three main directions. The number of elements and nodes used in the model were 4,510,203 and 6,668,053, respectively, and mesh convergence was achieved. According to the simulation in Figs. 1c and 1d, the maximum deformation was around 100 µm, and no von Mises stress concentrations were detected in the structure.

A two-part junction PLA layer comprised of a non-porous and a porous part was used to join the stiffer Ti matrix to a softer collagen–PLGA matrix. The non-porous PLA interlayer was used as a barrier to prevent synovial fluid from being “squeezed” into the underlying subchondral bone, and also to prevent bone marrow from invading into the cartilage layer. The 3D porous PLA lattice was designed with a strut diameter of 0.5 mm, a pore size of 0.5 mm and a pitch size of 1 mm to allow enough space for the penetration of the next layer of porous collagen, infiltrated with PLGA to improve the mechanical properties. The overall geometry of the osteochondral scaffold was designed as a truncated cone with 10-mm height, 6.35-mm lower diameter and 10-mm upper diameter to ease the surgical delivery procedure.

In the present study, a trilayered collagen–hydroxyapatite composite scaffold was used as control, as shown in Fig. 1b. This scaffold comprises of a top collagen layer for cartilage regeneration, a middle layer (60% collagen and 40% hydroxyapatite), and a bottom layer (30% collagen and 70% hydroxyapatite) for bone regeneration.

### Additive manufacturing of Ti matrix

**Fig. 2:**
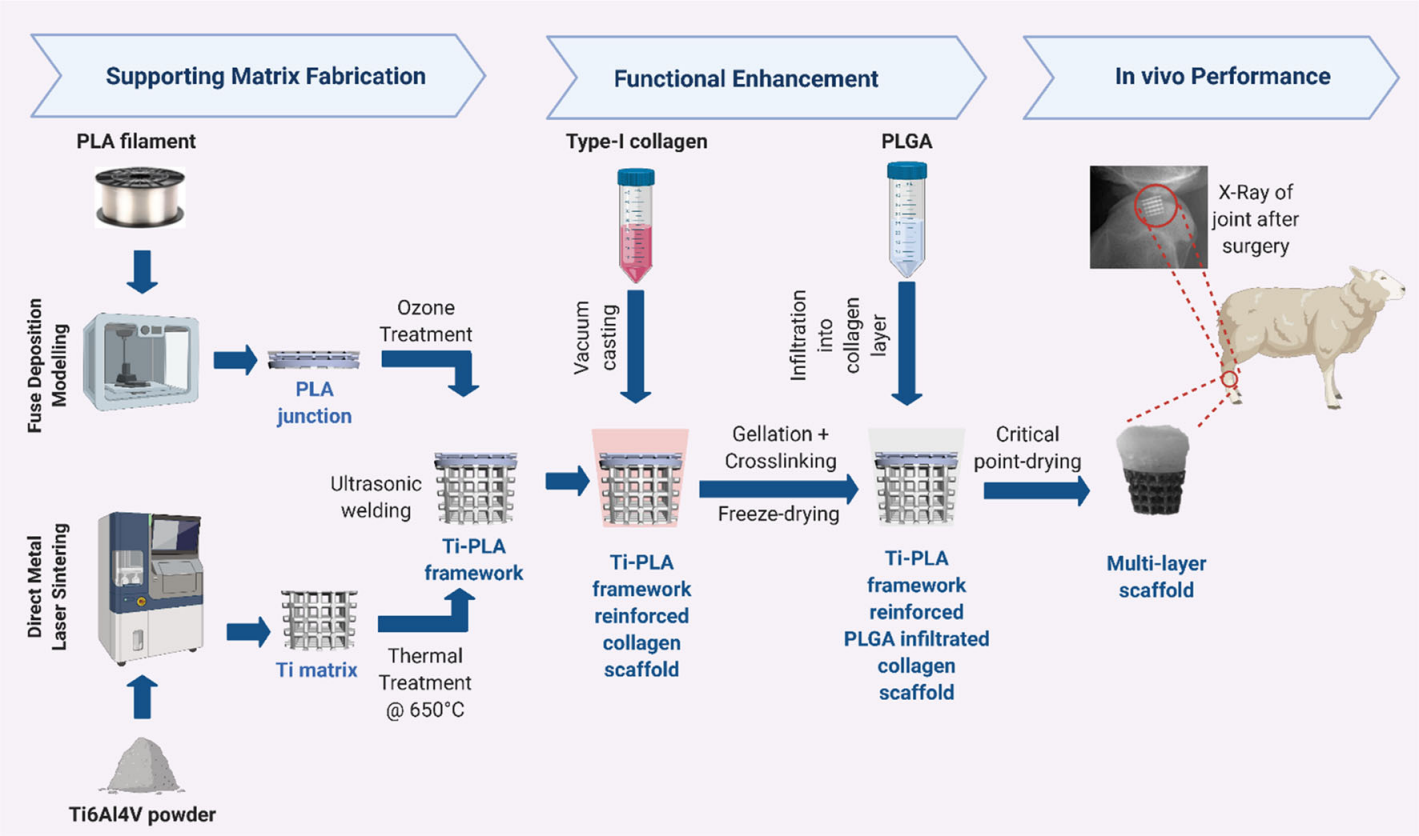
Schematic illustration of multi-layer osteochondral scaffold fabrication and in vivo study. The Ti matrix was manufactured by SLS and the PLA junction by FDM techniques, and they were joined by ultrasonic welding. Collagen was cast on the top Ti-PLA framework, crosslinked and freeze-dried. PLGA was then infiltrated into collagen to reinforce the matrix, and the construct was critical point dried. The efficacy was evaluated in a sheep stifle condyle for 12 weeks. Created with BioRender.com

Figure 2 shows the manufacturing flowchart and subsequent processing steps of the multi-layer scaffold. The Ti component corresponding to the bony part of the scaffold was manufactured from Ti6Al4V alloy using selective laser sintering (SLS) system for metal (EOS M270) in compliance with ASTM F2924 as previously described [24]. Briefly, 200WYb fibre laser was used to sinter the Ti powder. The scaffold was built at a speed of 4 mm^2^/s with layer thickness of 40 µm. The resultant Ti matrices were cleaned in an ultrasonic bath, dried, and thermally treated at 650 °C in an argon environment for 60 min to relieve any residual stresses.

### Fabrication of PLA junction layer

The PLA layer was fabricated using the fused deposition modelling (FDM) technique (FlashForge system). The 1.75-mm PLA (Verbatim) filaments were extruded through the 0.4-mm nozzle at 210 °C onto a 65 °C printing bed. The print and travel speed were both set to 20 mm/s, and the layer height was set to 0.25 mm. Two designs of the PLA layer were explored: (1) layers at 45° and (2) layers at 90°. The optimum design was decided upon in vitro characterisation, which showed that the design with layers at 90° better supported the cell proliferation. The experimental methods and results in terms of cell viability and proliferation are shown in Online Resource 1 in Supplementary Information. The optimal design was chosen for further in vivo analysis. To decrease hydrophobicity, the scaffold was subjected to UV/Ozone treatment (Bioforce, Procleaner Plus) for 1 min on each side. Subsequently, the 3D PLA layer was joined with the 3D porous Ti layer using ultrasonic welding to form a Ti matrix-reinforced PLA double-layered framework for further processing, as shown in Fig. 2.

### Collagen–PLGA layer

In order to prepare the cartilage layer, the Ti-PLA framework constructed as above was placed in a pre-designed mould. Type-I acid-soluble collagen (Collagen Solutions, UK, 6 mg/mL) was cast on top of the Ti-PLA framework in the mould and then crosslinked in situ using EDC/NHS as described previously [25]. The Ti-PLA–collagen assembly was then frozen at − 20 °C overnight and freeze-dried (Christ Alpha 1-2LD, UK). A 10% PLGA solution was prepared by dissolving PLGA powder (Resomer RG 858 S, Evonik, Germany) in acetone (VWR, UK). The collagen layer was infiltrated with PLGA solution, and the assembly was dried using a critical point dryer (Emitech K850, UK) (Fig. 2). The obtained osteochondral scaffold was 6.5 mm in lower diameter, 10 mm in upper diameter and 10 mm in height.

For the in vivo tests, a control scaffold was fabricated using type-I collagen and hydroxyapatite (HAp) (Sigma-Aldrich, UK) (collagen–HAp scaffold group). This control scaffold was chosen to be similar to a scaffold currently used in clinical trials. The collagen was processed in the same way as the multi-layer scaffold. The lower layer corresponding to bone was made from 30% collagen and 70% HAp, the mid-layer was made from 60% collagen and 40% HAp, and the top layer was pure collagen (Fig. 1b). The suspensions were placed layer by layer into a custom-made resin mould, frozen at − 20 °C and freeze-dried. Both types of scaffold were sterilised by gamma irradiation at 25 kGy (Synergy Health, UK).

### Scaffold characterisation

#### Evaluation of microstructure

The microstructure of layers was evaluated using scanning electron microscopy (SEM). The assembled scaffold was sectioned in a longitudinal direction using a low-speed precision diamond saw (IsoMet, Buehler, UK), mounted onto SEM stubs, coated with gold–palladium, and observed under SEM with an acceleration voltage of 5 kV. The geometrical parameters of Ti and PLA layers were determined from the SEM micrographs using Fiji/ImageJ [26], using measurements from Ti struts (*n* = 10), PLA filaments (*n* = 20) and Ti (*n* = 6), and PLA pore sizes.

The collagen–PLGA layer was also scanned using a Skyscan 1172 micro-CT with no filter at 47 kV and 145 µA to calculate the pore sizes. The 3D reconstructions were performed using NRecon software (v.1.6.3.2, Skyscan). The achieved resolution was approximately 4 μm per voxel in each axis (isotropic voxel). For a 360° scan, 2200 projection images were acquired. The pore sizes of collagen–PLGA scaffolds were determined using five (on average) projections per sample by Fiji/ImageJ [26]. A threshold was applied to each image, which was then despeckled and the watershed function was used to fit 2129 pores. The diameter was calculated from the area of the fitted pore.

#### Evaluation of mechanical properties

Axial compression tests were performed to evaluate the mechanical properties of the constructed layers under compression. The stiffness of the porous Ti part (*n* = 3, height = 6 mm, diameter = 10 mm) was measured using a Zwick Roell Z5 (Germany) material testing machine with a maximum load-bearing capacity of 5 kN and a crosshead speed of 1 mm/min. The load versus displacement was recorded, and the stress–strain curves were generated for each sample. The modulus of elasticity and compressive strength was calculated as per ASTM D695-02 [27].

The PLA layer (height = 6.5 mm, diameter = 14.3 mm) was tested under compression using a Sans Universal Testing Machine (Shenzhen, China) with a maximum load-bearing capacity of 10 kN and a crosshead speed of 0.65 mm/min according to ASTM D1621-16. The compressive modulus and strength were calculated from the load–displacement curves following the instructions of ASTM D1621-16.

The compressive properties of the collagen–PLGA layer (height = 7 mm, diameter = 7 mm) were measured using a Bose ElectroForce BioDynamic (TA Instruments, USA) device with a maximum load-bearing capacity of 200 N and a crosshead speed of 0.5 mm/min in the dry state to obtain the stress–strain curves. The compressive modulus was calculated from the linear regression of the initial linear regime of the curve, and the compressive strength was determined from the intersection of the elastic and plateau regions of the regression curves [28].

The adhesion strength between the Ti and PLA layers was determined using a modified shear test (Emco F1 CNC). The Ti part of the sample was held in a custom-made holder, while the compressive force was applied to the PLA layer using a custom-made curved endcap at a rate of 2 mm/min. The load at failure was recorded.

### Cell-scaffold interactions

The biocompatibility of the multi-layer scaffold was evaluated using sheep bone marrow mesenchymal stem cells (sBMMSCs). These cells were isolated from sheep bone marrow aspirate, expanded and maintained in tissue culture flasks containing complete media (Dulbecco’s Modified Eagles Medium—DMEM, Sigma-Aldrich, UK) supplemented with 10% foetal calf serum (FCS, First Link, UK) and 100 units/mL of penicillin and streptomycin (Gibco, UK). The flasks were kept at 37 °C with 5% CO_2_, and passaged when 80% confluency was reached. The sBMMSCs were characterised through demonstrating their multipotency by differentiating them down the adipogenic, chondrogenic, and osteogenic lineages. Cells of passage 3–5 were trypsinised and seeded with a concentration of 1 × 10^6^ cells per layer. For the in vitro tests, each sample was sterilised by incubation in 70% ethanol for 15 min, subsequently washed with sterile phosphate saline buffer (PBS, Gibco, UK) and incubated in complete medium. Prior to cell seeding, the samples were blot-dried with sterile filter paper and placed in untreated 12-well plates (Fisher Scientific, UK). The cells were allowed to adhere to the scaffold for 1 h before the addition of 2-mL complete medium to each well. The samples were then incubated for up to 14 days, and the medium was replaced every 2–3 days.

The cell viability on the scaffold layers was evaluated using live/dead assay on days 1, 3 and 7. For this assay, the samples were incubated with 1 mL of live/dead reagent (15 µL of Calcein AM and 17 µL of ethidium homodimer in 5 mL of PBS) for 45 min at 37 °C and 5% CO_2_. They were then rinsed with PBS, mounted on glass slides and observed under an Apotom2 Zeiss fluorescent microscope.

The alamarBlue™ (Thermo Fisher, UK) cell viability reagent was used to examine the proliferation of cells on the samples on days 1, 7 and 14. The samples were tested in triplicate, and one sample with no seeded cells was used as control. At each time point, the samples were moved onto a new plate, and 1.5 mL of media containing 10% alamarBlue was added to the well. The samples were incubated for 4 h, and then 200 µL of supernatant was transferred to a black 96-well plate. The readings were taken by a Tecan Infinite M200 Pro microplate reader at 530–560 nm excitation and 590 nm emission.

### In vivo evaluation using ovine stifle condyle model

The aim of this study was to evaluate the short-term safety and efficacy of the multi-layer osteochondral scaffold (12 weeks). This arrangement is compliant with the recommendations of ISO 10993–6:2016 Biological evaluation of medical devices—Part 6: tests for local effects after implantation. Our choice of the control group was based on the principles of replacement, refinement or reduction in the use of animals (3Rs), the UK Home Office regulations on animal research and the recommendation of the University’s Animal Welfare and Ethics Committee (AWERB). We opted to use a scaffold similar to a commercially available osteochondral scaffold of collagen/hydroxyapatite (collagen–HAp) instead of an empty defect. The reason was firstly to avoid sacrificing more animals when it is known that these empty defects will not fully repair, and secondly to show if there is any clinically and commercially significant enhancement over current products.

In vivo assessments were carried out in the sheep medial femoral condyle under the approval of and in compliance with the UK Home Office requirements of the Animals (Scientific Procedures) Act 1986, which included local ethical approval by the Royal Veterinary College Ethics Committee. A total of ten skeletally mature, adult female sheep (Royal Veterinary College, UK) of 80.7 kg mean weight (72–88 kg) were used in the study. Anaesthesia was induced with a mixture of ketamine and midazolam and then maintained using gaseous isoflurane at 2.5% with oxygen. The sheep were administered Ceporex antibiotic injections preoperatively and on days 1, 2 and 3. In addition, each sheep had fentanyl patches applied to a shaved area on the forelimb one day before surgery. This patch was replaced 3 days after surgery.

Under anaesthesia, an incision was made on the left limb using the medial parapatellar arthrotomy approach to obtain access to the femoral condyle. A conical critical-sized OC defect matching the size of the scaffold was created using custom-made surgical drills on the load-bearing area of the medial femoral condyle. The animals were randomly assigned to the collagen–HAp scaffold group (*n* = 4) or the multi-layer scaffold group (*n* = 6). In the collagen–HAp group, the defects were filled with the collagen–HAp scaffold, while in the multi-layer group, the multi-layer scaffolds were press-fitted into the defect until the top of scaffolds were levelled with the native cartilage surface. The animals were housed in individual pens for 4 (± 1) days after surgery and then transferred to a group pen for the remainder of the study (12 weeks).

At the time of implantation, the scaffolds were filled with bone marrow and blood that was generated at the bone-implant interface, and they were inserted successfully into the defect using the press-fit implantation technique. After the operation, all animals recovered well, and they were able to move freely within 5 days. A slight limp was observed in one of the animals, which recovered after a few days. No post-operative complications were observed up to the end time point.

The gaits of the sheep were monitored, and their limbs were scanned radiologically after implantation and before euthanasia at 12 weeks. The condyles of both legs were harvested after 12 weeks for further analysis. They were kept in phosphate saline buffer (pH 7.4) until peripheral quantitative computed tomography and then preserved in 10% neutral buffered formalin (Thermo Scientific, UK) for further examination.

### Macroscopic assessment

The sheep were euthanised at 12 weeks, and the joints were opened to examine the defect site and surrounding joint tissues. Photographs of the defect sites were taken, and an assessor who was blinded to the type of implant evaluated the quality of cartilage repair and degree of regeneration looking at five different areas of comparison (cartilage colour, homogeneity, surface smoothness, lateral integration, and defect filling) with the maximum score of 10 for each (10 = normal cartilage tissue). The scoring system for macroscopic evaluation is described in the table in Online Resource 2 in Supplementary Information.

### Peripheral quantitative computed tomography (pQCT)

A pQCT (Stratec XCT 2000, Germany) instrument was used to scan the specimens and analyse the volumetric bone mineral density (vBMD, mg/cm^3^) distributions. Each condyle was placed in a plastic holder, subsequently positioned in the scanner of the pQCT and scanned in four planes starting from the middle of each sample with steps of 1 mm, voxel size of 0.2 mm × 0.2 mm and scan speed of 20 mm/s. The analysis of vBMD was performed by the XCT3000 (2000) software (version 6.20, Stratec, Germany). In each slice, a region of interest surrounding the defect was defined for the measurement of trabecular bone mineral density (CALCBD). This was then normalised to the vBMD value of the unoperated leg (right leg) of each sheep and reported as relative vBMD. This normalisation was applied to reduce the effect of variability between the sheep individuals.

### Micro-computed tomography (micro-CT) analysis

The samples were scanned with a Nikon XT H 225 at 70 kVP and 112 mA (resolution of about 8–18 µm) to visualise the newly formed bone tissue within the defect site. Three-dimensional reconstructions were performed using Nikon XT software, and subsequent visualisation was conducted using Bruker Software CTVOX.

### Histological analysis and histomorphometry

The sheep knees were collected, and the medial femoral condyles were excised for histological analysis. A 2-mm diameter biopsy punch (Kai Medical, Germany) was used to collect a cartilage core from the regenerated tissue, which was processed for wax histology. Sections with 8-µm thickness were collected from each sample and stained using routine H&E, Safranin-O and Alcian Blue stains.

The presence of collagen type-II was detected as previously described [29] using primary polyclonal rabbit antibodies against collagen-II (Abcam, ab34712) and a secondary goat anti-rabbit IgG conjugated to Alexa Fluor 488 (Abcam, ab150077).

Following deparaffinisation, rehydration and enzymatic antigen retrieval using pepsin (equilibration solution (0.02 M HCl) for 10 min, and pepsin digestion solution (10 mg/mL in 0.02 M HCl) at 37 °C for 45 min in a humidified chamber), the slides were blocked with 5% (v/v) goat serum in 0.01% (v/v) Triton X-100/PBS for 60 min. Subsequently, the primary (1:200) and secondary antibodies (1:200) were added to the slides. They were mounted with Fluoroshield containing DAPI (Sigma-Aldrich, UK) and stored at 4 °C in the dark. Slides were then imaged with a Zeiss Apotome 2 fluorescence microscope (Zeiss, Germany). One blinded assessor scored the samples using the modified ICRS II scoring system [30]. To analyse the bone and the implants for histomorphometry, the condyles were dehydrated through a series of alcohol treatments and transferred to LR White Resin (London Resin Company, UK). The resin was set using an accelerator according to the manufacturer’s recommendation. Undecalcified 300-μm-thick sections along the long axis of all condyles were cut in a parallel direction using a diamond saw micro-sectioning system (Exakt Apparatebau, Norderstedt, Germany). These sections were reduced to 100-μm thickness and polished on a Motopol 2000 apparatus (Buehler, Coventry, UK). Before performing histological analysis, the sections were imaged using BioVision + radiography equipment (Faxitron, USA) to visualise the mineralised tissue and bone. Prior to histomorphometric analysis (trabecular bone volume, %BV/TV), the sections were stained as previously described [31] with toluidine blue [32] that stains fibrous tissue blue, and Paragon [33] that stains new bone tissue bright pink for 20 min each. The sections were imaged with a Zeiss Apotome 2 fluorescence microscope (Zeiss, Germany). A high-magnification snapshot was imported into ImageJ [34] and thresholded to distinguish the bone and non-bone tissues. The %BV/TV was calculated as the area of bone to the sum of areas of bone and non-bone tissues.

### Quantitative reverse transcription-polymerase chain reaction (qRT-PCR)

The cartilage-related markers aggrecan (ACAN) and collagen type-II (COL II) were analysed using qRT-PCR. Immediately after euthanasia, 2-mm biopsies of the regenerated cartilage were harvested and transferred to RNAlater™ Stabilisation Solution (Invitrogen, UK). TRIzol reagent (Invitrogen, USA) was used to extract the total RNA by following the manufacturer’s recommendations. cDNAs were synthesised using SuperScript VILO cDNA kit (Thermo Scientific, UK), and qRT-PCR reactions were carried out with a Biorad CFX96 Real-Time PCR Detection System (Bio-rad, UK) using the Brilliant III SYBR Green qPCR Master Mix kit (Agilent, California, USA) in accordance with the manufacturer’s instructions. The primers for the targeted genes and the internal control gene β-microglobulin were purchased from Sigma-Aldrich (UK) as listed in Online Resource 4 in Supplementary Information. Normal, healthy cartilage was used as control, and relative gene expression was quantified using the ΔΔ*C*_T_ method [35]. The statistical analysis was performed on ΔΔ*C*_T_ values, while the 2^−∆∆*C*^_T_ values were used to calculate the mRNA expression fold change.

### Statistical analysis

The statistical analysis was performed using Graphpad Prism 8.1 and OriginPro 2019 software. All quantitative data were expressed as mean ± standard deviation unless specified otherwise. A Shapiro–Wilk test was used to determine the normality of data. For each normally distributed parameter, statistical analysis was performed using student’s *t* test or one- or two-way ANOVA with Sidak post hoc test, and a value of *p* < 0.05 was considered as statistically significant. A Mann–Whitney U test was performed for data that were not normally distributed.

## Results

### Scaffold characterisation

**Fig. 3:**
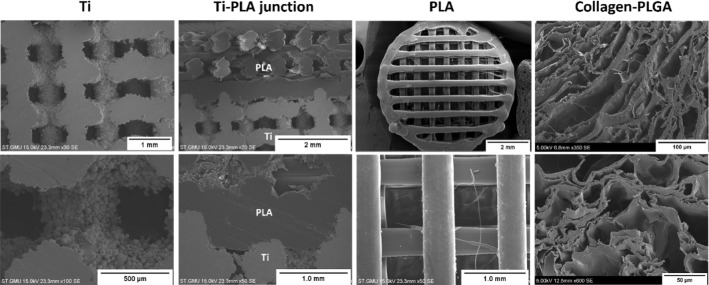
SEM micrographs of the cross section of scaffold and the individual layers

The microstructure of the multi-layer scaffold was examined using SEM, as shown in Fig. 3. The cross-sectional examination of the scaffold revealed the overlapping of the PLA and Ti layers, forming a secure fixation of the polymer onto the metal matrix.

**Fig. 4:**
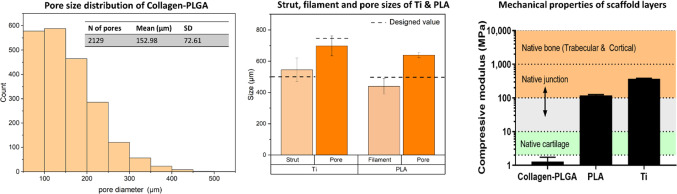
Microstructural evaluation of the scaffold layers; the pore size distribution of the collagen–PLGA layer showed that the majority of pores reside within 150–250 µm. The strut, filament and pore sizes of Ti and PLA layers were determined by SEM micrographs and compared with the designed value. The compressive modulus of layers are in the region of the native tissue, which corresponds to values of both the trabecular and the cortical bone of femur; the values were taken from the literature [37, 38]

Table 1 and Fig. 4 describe the strut, filaments and pore sizes of the Ti and PLA layers calculated from the SEM micrographs.

**Table 1:** Struts, filaments and pitch sizes measured after printing (*n* = 6–15)

Layer	Strut/filament diameter (µm)	Pore size (µm)
Ti	545.4 ± 75.6	698.2 ± 63.4
PLA	440.4 ± 49.1	639.01 ± 16.5

It was observed through SEM examination that the strut sizes of Ti were not significantly different from the design value of 750 µm, and the actual pore sizes seemed to be smaller than that of design value. In the case of PLA, the filaments were printed thinner compared to the design value of 500 µm, namely 440.4 µm (± 49.1). It was also observed by the SEM examination that the collagen–PLGA showed a highly interconnected porous architecture, and its pore sizes were quantified using the images from micro-CT. Figure 4 presents the histogram of pore diameter distribution in collagen–PLGA. It was revealed that the pores had a mean diameter of 152.98 µm (± 72.61), and the majority of pores were in the range of 150 to 250 µm, which are best-suited for cartilage formation [36].

The mechanical properties (modulus and strength) of each layer under compression in the dry state were tested, as reported in Table 2 and Fig. 4. As observed, the compressive strengths were within the range of the mechanical properties of normal osteochondral tissue. The adhesive strength between the Ti and PLA layers was examined, and the maximum load to failure and adhesion strength were calculated to be 105.5 ± 19.7 N and 1.34 ± 0.25 MPa, respectively. Interestingly, the failure occurred between the layers of PLA and not at the junction of Ti and PLA.

**Table 2:** Mechanical properties of scaffold layers tested under compression

Component	Compressive strength (MPa)	Modulus of elasticity
Ti matrix	63.14 ± 2.16	0.37 ± 0.01 (GPa)
PLA junction layer	7.33 ± 0.32	118.9 ± 6.25 (MPa)
Collagen–PLGA	0.21 ± 0.04	1.28 ± 0.45 (MPa)

The results are expressed as mean ± standard deviation

### In vitro biocompatibility

**Fig. 5:**
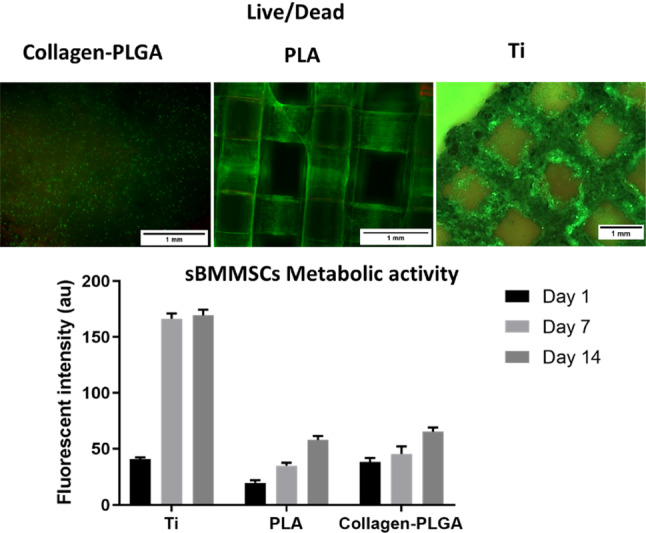
Cell-scaffold interaction: the viability and proliferation of sBMMSCs on the layers of scaffold. Live/dead staining shows mostly viable cells (green) on day 14; the alamarBlue assay shows cell proliferation over 14 days

The in vitro assays performed on collagen–PLGA, PLA and Ti layers confirmed their biocompatibility. The live/dead results, shown in Fig. 5, show the viability of cells throughout 14 days of culture. Most of the cells were alive in all of the layers, as demonstrated by the green colour. The cells seemed to distribute throughout the samples, and most of the surface of PLA and Ti were covered after 14 days.

The proliferation of sBMMSCs was evaluated quantitatively using the alamarBlue assay, with the results shown in Fig. 5. The proliferation analysis revealed that all layers supported cell proliferation, as confirmed by a continuous increase in alamarBlue activity over 14 days. The higher cell proliferation on Ti scaffolds was linked to their higher surface area available for cell growth when compared to the other samples.

### Cartilage regeneration in the osteochondral scaffold

**Fig. 6:**
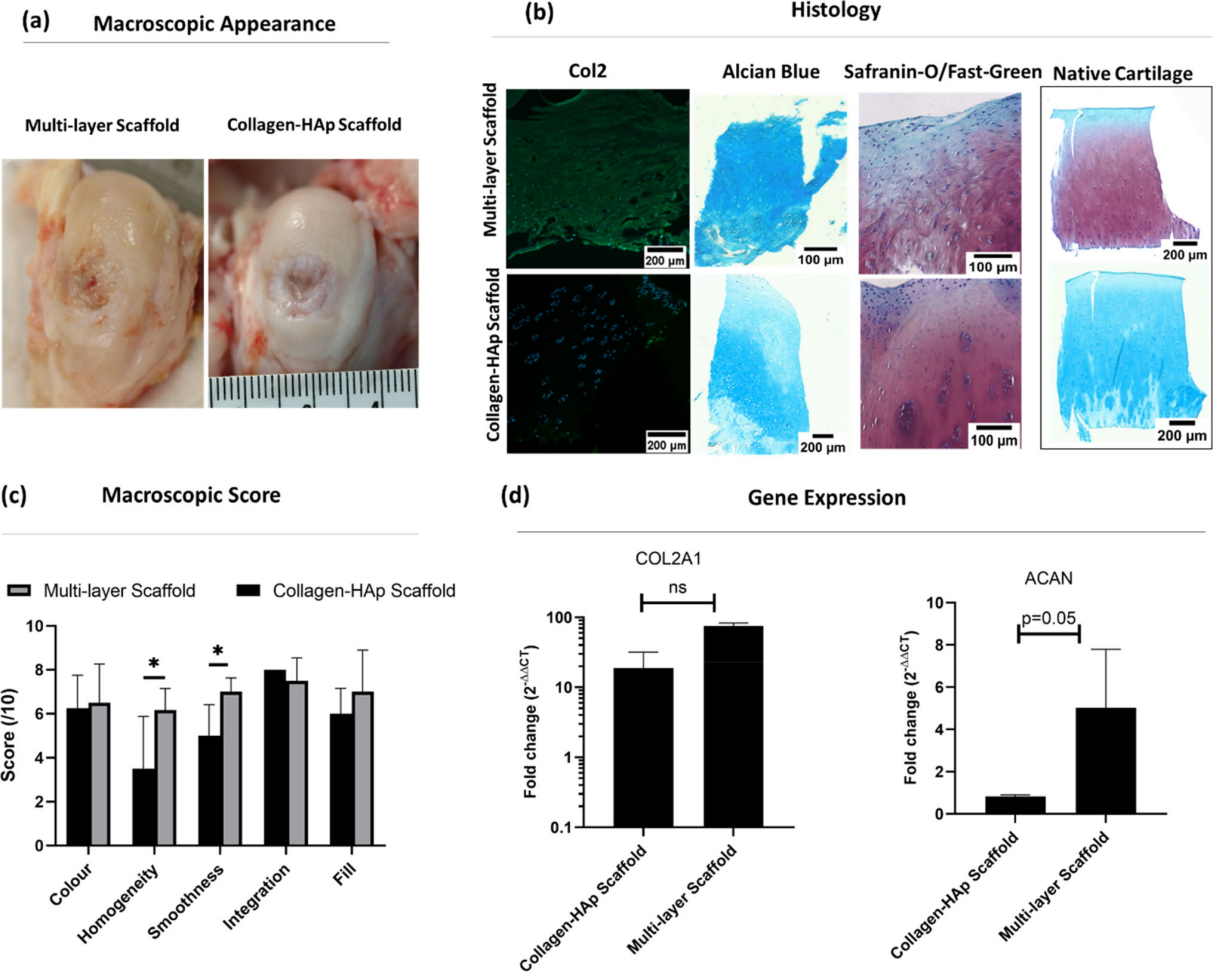
Assessment of the regenerated cartilage. **a** Visual assessment of the regenerated cartilage in the multi-layer scaffold and collagen–HAp scaffold groups after 12 weeks. **b** Histological staining of cartilage with Alcian Blue, Safranin-O and collagen II/DAPI in the multi-layer scaffold and collagen–HAp scaffold groups, as well as in native cartilage. **c** Gross morphological scores for repaired cartilage in the multi-layer scaffold and collagen–HAp scaffold groups; newly formed cartilage was significantly smoother and more homogenous in the multi-layer scaffold group. (^*^ significance at *p* = 0.03 & *p* = 0.01). **d** Gene analysis for chondrogenic-related genes at week 12. The qRT-PCR results demonstrate that the multi-layer scaffold group showed a higher expression of cartilage-associated genes, especially aggrecan (ACAN), which had borderline significance (*p* = 0.0507). The error bars represent the standard error of the mean

After opening the joints, the gross macroscopic appearance of the repair tissue was evaluated; the macroscopic assessment is presented in Fig. 6a. Based on visual assessment, we did not observe degenerative changes or delamination of the multi-layer scaffold in the joints.

At 12 weeks, enhanced levels of defect fill were observed in both the collagen–HAp scaffold and the multi-layer scaffold groups. However, in the collagen–HAp scaffold group, the central region of the defect was consistently unfilled, as opposed to the multi-layer scaffold group where a more homogenous response was observed. Adequate integration of the repair tissue with the surrounding tissue was observed in both groups; however, in the collagen–HAp scaffold group, the smoothness of the repaired tissue was consistently lower than that in the multi-layer scaffold group, featuring large cracks and fissures, as shown in Fig. 6a. The colour of the regenerated cartilage was similar to that of the surrounding native tissue, especially in the periphery. In contrast, in the collagen–HAp scaffold group, the central area remained red. In the multi-layer scaffold group, the location of the defect seemed to have certain effects on the morphology of the repair tissue. Overall, as shown in Fig. 6a, when the defects were more central to the condyle, better fill, colour, and homogeneity were observed compared to a defect on the edge, which is considered to be due to the difference in mechanical forces in those regions.

The gross morphological scores, as shown in Fig. 6c, were in accordance with the visual findings. The homogeneity and smoothness of the repaired cartilage were significantly (*p* = 0.03 and 0.01, respectively) better in the multi-layer scaffold group, indicating that the gross morphological appearance of the regenerated cartilage was superior in this group compared to the collagen–HAp scaffold group.

In addition to the gross morphological evaluations, histological and immunohistological assessments were performed, which confirmed that the multi-layer scaffolds were able to improve the repair of articular cartilage relative to the collagen–HAp scaffolds. The histological staining of both groups showed evidence of newly formed repair tissue that integrated with the surrounding native tissue. The staining of the biopsy taken from the repair cartilage with Safranin-O indicated hyaline-like cartilage formation, and a more intense and uniform Alcian Blue stain was observed in the multi-layer scaffold group compared to the collagen–HAp scaffold group. Cells within the repair cartilage were seen to reside within the lacunae and showed a rounded morphology. The collagen type-II staining of these tissues confirmed the formation of hyaline-like cartilage in the multi-layer scaffold group (Fig. 6b).

The histological staining of newly formed cartilage was quantitatively scored using a modified ICRS II scoring system. This scoring system is described in the table of Online Resource 3 in Supplementary Information. The multi-layer scaffold group demonstrated improved repair and a higher quality of repair tissue, as shown by the higher histological scores (72 vs 62) compared to the collagen–HAp scaffold group, although the difference was not significant (*p* > 0.05). A significantly higher chondrocyte clustering (*p* = 0.006) was observed in the collagen–HAp scaffold group, a condition that is linked with osteoarthritic tissues.

Chondrogenesis was evaluated by looking at the relative expression of aggrecan (ACAN) and collagen II (COL II) in the multi-layer scaffold and collagen–HAp scaffold groups compared to healthy cartilage using qPCR at 12 weeks (Fig. 6d). The upregulation of both of these chondrogenic genes was observed in the multi-layer scaffold group, while ACAN was downregulated in the collagen–HAp scaffold group. This difference was not significant for COL II (*p* = 0.15); however, it had borderline significance for ACAN (*p* = 0.0507).

### Subchondral bone regeneration

**Fig. 7:**
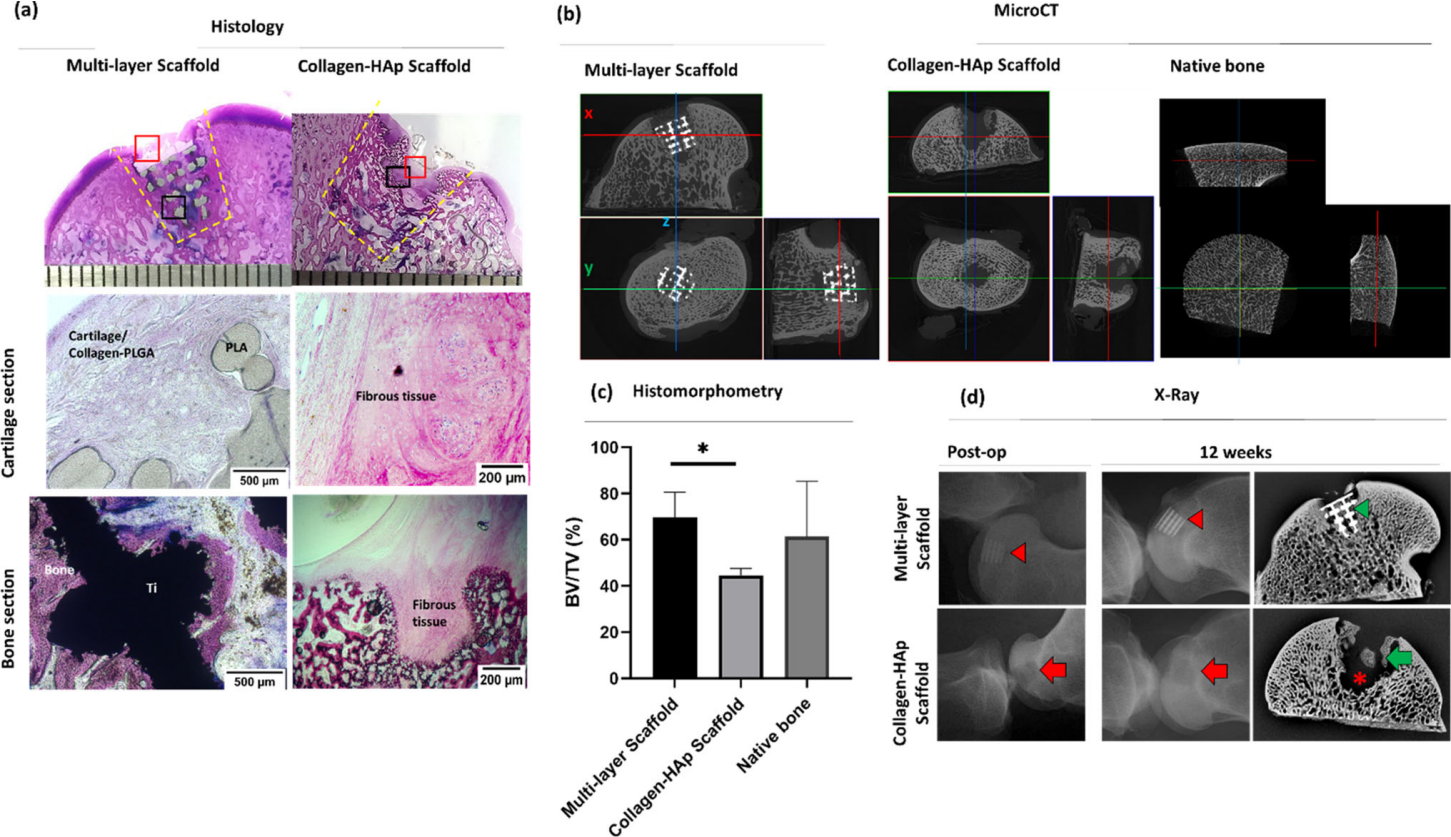
Bone regeneration: **a** Undecalcified histology of the multi-layer scaffold and collagen–HAp scaffold groups show bone formation surrounding the Ti matrix and tissue ingrowth into the PLA section, while fibrous tissue formation is observed in the collagen–HAp scaffold group. **b** The micro-CT sections of samples in both groups clearly show the presence of voids in the collagen–HAp scaffold group. **c** The histomorphometry analysis from histology showed significantly higher (*p* = 0.01) bone volume/total volume (bone ingrowth) in the multi-layer scaffold group. The BV/TV of native bone taken from the literature [39]. **d** Evaluation of bone formation. The X-ray micrographs of mid-sections of both groups and the X-ray of the joint immediately after the operation and at 12 weeks showing multi-layer scaffold and collagen–HAp scaffold samples. The red asterisk shows the lack of bone formation in the collagen–HAp scaffold group, with only patches of new bone (green arrow). The green arrowhead shows bone ingrowth into the Ti matrix. The red arrowhead indicates the position of the scaffold and its fixation within the joint. The area shown by the red arrow depicts the position of the collagen–HAp scaffold (note the reduced opacity)

The X-ray examination of the limb was performed immediately after implantation and 12 weeks post-operation to assess the stability of scaffolds in the joint. The X-ray micrographs of the multi-layer scaffold group and collagen–HAp scaffold group are shown in Fig. 7. It was observed that the multi-layer scaffolds achieved a stable mechanical fixation to the surrounding bone, probably as a result of bone ingrowth into the porous structure of Ti. On the other hand, in the collagen–HAp scaffold group, the bone defect created during the operation was unfilled and the quality of regenerated tissue was different from its surroundings, as depicted by the difference in bone opacity.

**Fig. 8:**
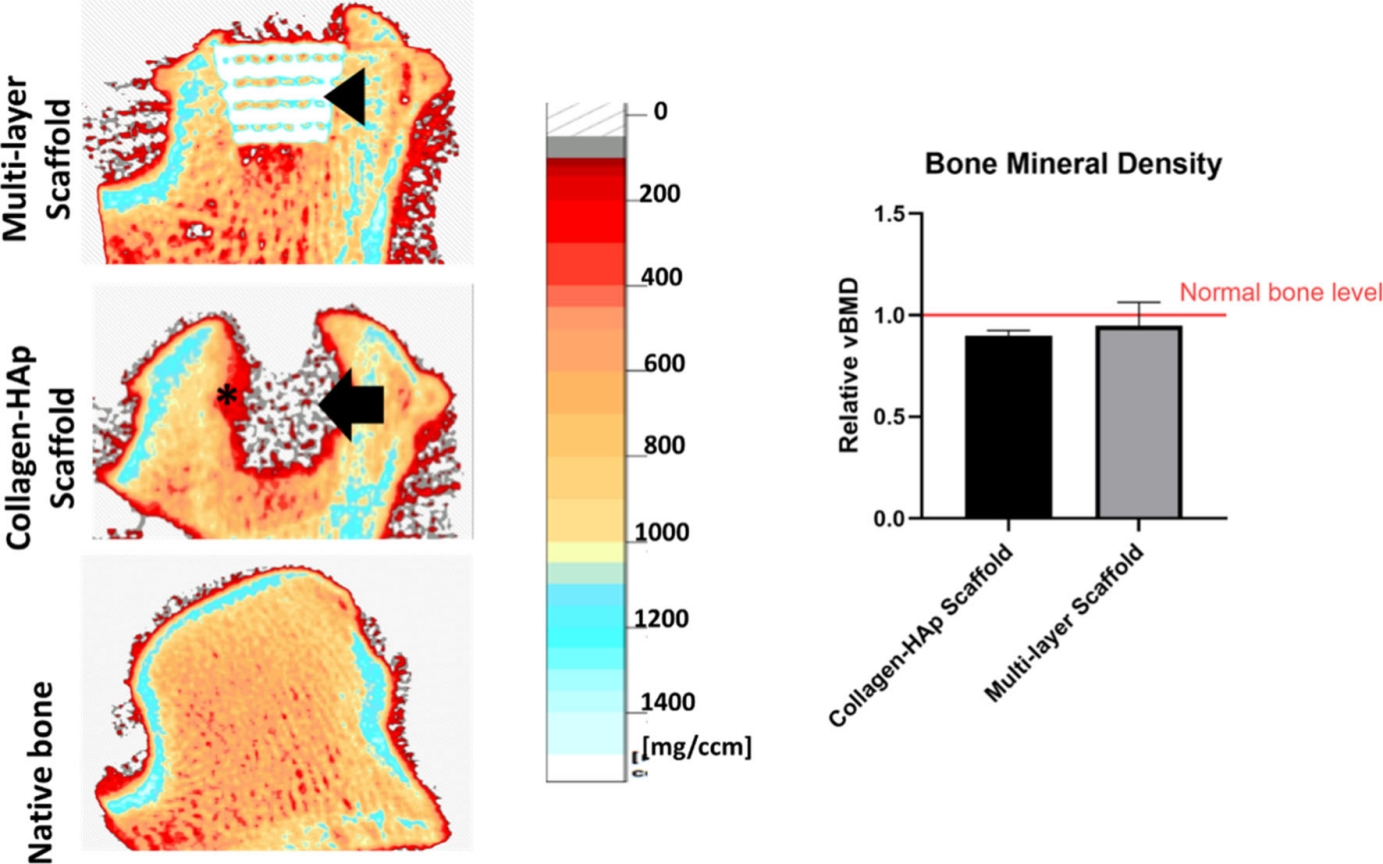
Volumetric bone mineral density of the repaired tissue and the surrounding bone, as measured by pQCT, with the multi-layer scaffold group showing a higher vBMD, which is more like that of the native bone (*p* > 0.05). The normalised values show the values of the regenerated bone in the operated leg to those of the unoperated leg of the same sheep. The black arrowhead denotes the Ti and the surrounding bone with a normal vBMD in the multi-layer scaffold group, whereas the black arrow denotes a non-mineralised/void area in the middle of the condyle in the collagen–HAp scaffold group with the resorbing surrounding bone depicted by the asterisk

The newly formed bone was characterised by undecalcified histology and micro-CT evaluation. The histology examination of the retrieved tissues, shown in Fig. 7, demonstrated extensive bone ingrowth into the porous Ti structure in the multi-layer scaffold group, inducing a good mechanical fixation to the surrounding tissue. In contrast, insufficient subchondral bone regeneration was observed in the collagen–HAp scaffold group, and the newly formed tissue was fibrous-like (Fig. 7a). These findings were also confirmed by X-ray examination of the histology Sects. 12 weeks post-operation, as reported in Fig. 8, which indicated that bone resorption occurred around the collagen–HAp scaffold. To further visualise bone regeneration, micro-CT examination was applied to the retrieved tissues (Fig. 7b). It was observed that the bone volume ratio (BV/TV) reached 68% in the multi-layer scaffold group. This result was significantly higher (*p* = 0.01) than that for the collagen–HAp scaffold group, which showed a BV/TV value of 45% (Fig. 7c). The bone-implant contact was calculated to be 61% (± 15%).

Similar to the above, volumetric bone mineral density (vBMD) for trabecular bone surrounding the defect was quantified using pQCT. Considering the variation between the sheep individuals, the vBMD of the operated leg was normalised to the unoperated leg of the same sheep to reduce the effects of such variations. Consistently higher values of bone mineral density in the multi-layer scaffold group compared to the collagen–HAp scaffold group (*p* > 0.05) were observed. While the vBMD of the bone in the multi-layer scaffold group resembled that of the native healthy bone, as seen from the relative vBMD of near 1 in Fig. 8, the regenerated bone in the collagen–HAp scaffold group, especially in the centre of the defect, seemed to be of relatively low mineral density.

## Discussion

Additive manufacturing techniques can potentially address the challenges associated with the fabrication of tissue engineering scaffolds for regenerating large osteochondral defects, giving more control over the choice of material, complex anatomical geometry and internal structure of the osteochondral scaffold. Although biofabrication techniques have been increasingly explored in research settings, few viable regenerative products for the routine clinical treatment of osteochondral defect have reached the early stage of OA. This is in part due to translational barriers to take an innovation from “discovery” in the laboratory to “translation” in the clinic. For more matured technologies and materials, a greater volume of data is available regarding their history of safe use, which in turn increases the probability of them being translated into routine clinical care. In order to fabricate an osteochondral scaffold that is closer to the “translation” stage in the clinic, we took advantage of biofabrication technologies and materials that are more established, i.e. we used laser sintering to fabricate the porous Ti layer corresponding to the bone, and material extrusion to fabricate the PLA layer corresponding to the bone-cartilage junction.

Osteochondral defects are often associated with the mechanical instability of the joint, and thus increase the risk of generating osteoarthritic degenerative changes [40]. In fact, by examining the current available osteochondral scaffolds registered on ClinicalTrials.gov (including completed and ongoing clinical trials), it was found that one of the main reasons for their failure in achieving the clinical satisfactory outcome is their insufficient bone integration, resulting in newly regenerated cartilages lacking the underlying biomechanical support of the subchondral bone. Without this strong biomechanical support, these cartilages will be lacking appropriate mechanical stimulation, which is a key factor for hyaline cartilage healing. As a result, fibrocartilage is often observed instead of hyaline cartilage, which has inferior quality and durability, and will not be long-lasting. Therefore, the main focus of the proposed osteochondral scaffold was to simultaneously enhance the repair and regeneration of cartilage and subchondral bone, so that the enhanced subchondral integration can help to achieve stable mechanical fixation of the scaffold and provide strong support for the overlying cartilage.

Compared to osteochondral scaffolds proposed in other studies [41–43], our multi-layer osteochondral scaffold showed superior mechanical performance in terms of both compressive modulus and compressive strength, with the values in the range for natural osteochondral tissue. In addition, the biocompatibility of the multi-layer scaffold was confirmed by in vitro evaluation with respect to the viability and proliferation of sBMMSCs.

Aimed at assessing the efficacy of the scaffold in the regeneration of large osteochondral defects, an in vivo sheep experiment targeting the load-bearing region of the femoral condyle was performed, and the results were collected after 12 weeks to give us a glimpse of its performance in the short term. The results showed enhanced repair of the articular cartilage and the corresponding subchondral bone in the multi-layer scaffold compared to a conventional collagen–HAp scaffold (Figs. 6–8).

The micro-CT and histological analyses showed an extensive amount of bone formation throughout the porous Ti layer of the multi-layer scaffolds at 12 weeks after implantation. There was a good integration of this newly formed bone with the neighbouring native bone, which likely contributed to the secure attachment of the implant within the defect. The subchondral bone is key to the regeneration of healthy articular cartilage, since inadequate repair of the subchondral bone will affect the biomechanical properties of the whole osteochondral region and negatively influence the longevity of the repair tissue [44–46]. In fact, the voids in the subchondral bone area of the collagen–HAp scaffold samples (Figs. 7 and 8) resemble the cavities in the subchondral bone usually observed in OA patients, referred to as “subchondral bone cysts” [47], and those of “unfilled bone voids” observed in some clinical scaffolds (e.g. TruFit [13, 14, 48], MaioRegen and Chondromimetic). It is therefore reasonable to assume that the presence of these cysts can lead to changes in the loading of joint and affect the quality and durability of regenerated cartilage. In natural cartilage, the external stresses exerted during physiological loading induce hydrostatic pressure that supports the load. This may be a function of the subchondral bone, which is not permeated by any blood vessels in adults and possibly contributes to maintaining this hydrostatic pressure and supporting the cartilage. Changes in the properties of the subchondral bone interfere with this function, and can consequently lead to increased levels of strain in the cartilage layer, which aids the initiation of matrix destruction [49]. This may be the reason for the inferior regeneration of cartilage in the collagen–HAp scaffold group in our study. The regenerated cartilage in the multi-layer scaffold group was hyaline-like and superior to that found in the collagen–HAp scaffold group, as demonstrated by histological analysis (Fig. 6b). The gene expression analysis of the newly synthesised cartilage also showed improved chondrogenesis in the multi-layer scaffold, as compared with that in the collagen–HAp scaffold (Fig. 6d). It has been shown that early bone integration supports the regeneration of the overlying cartilage [44], and these findings prove that a mechanically stable osteochondral scaffold has the same effects as early bone repair, improving the quality of regenerated cartilage.

With regard to the degradability of the multi-layer scaffold components, the Ti matrix will remain in the joint in the long-term, while collagen–PLGA and PLA matrices degrade over time. Although this non-degradability could be clinically unfavourable for the treatment of small defects, the support it provides may be required for large defects. The present study illustrated the short-term performance of the scaffold, while longer-term studies (6 months [50]) will be necessary to confirm the durability of our results.

The scaffold developed herein combines a more “translation-ready” technology with improved efficacy is intended for the treatment of large osteochondral defects, and is expected to lead to tangible and clinically relevant results in a one-step surgical procedure.

## Conclusions

In this study, a novel multi-layer osteochondral scaffold for the repair and regeneration of large osteochondral defects has been successfully developed by using techniques and materials that are closer to the “translation” phase of additive manufacturing technologies. The in vivo evaluation of the developed scaffold in sheep stifle condyle demonstrated that there were no adverse effects during the 12-week evaluation period, and the multi-layer scaffold achieved a stable mechanical fixation owing to the improved bone ingrowth into the porous structure of the Ti matrix. Based on these initial short-term results, it seemed that the bone surrounding the Ti matrix had a higher bone mineral density in the multi-layer scaffold group than that in the collagen–HAp scaffold group, which provided strong support to the overlying cartilage, leading to enhanced cartilage fill. As confirmed by histology examination, the newly formed cartilage was hyaline-like tissue. This multi-layer scaffold technology has shown the potential to address the unmet clinical need for the repair of large chondral and osteochondral defects in the early stage of OA, and will hopefully provide clinicians with a viable treatment option in situations where the disease has progressed beyond a small defect, but a full joint replacement can still be avoided.

## Supplementary Information

Below is the link to the electronic supplementary material.

Supplementary file 1 (DOCX 620 kb)

## Declarations

### Availability of data and materials

Available upon reasonable request from the corresponding author.
