# Supplementary material for: In vivo evaluation of additively manufactured multi-layered scaffold for the repair of large osteochondral defects
Source: Biodes Manuf. 2022 Mar 16;5(3):481–96. doi: 10.1007/s42242-021-00177-w (PMC9279224; doi:10.1007/s42242-021-00177-w)
Supplement: Supplementary file 1 — (DOCX 620 kb) [file 42242_2021_177_MOESM1_ESM.docx]

# Online Resources – Bio-Design and Manufacturing

**In vivo evaluation of additively manufactured multi-layered scaffold for the repair of large osteochondral defects**

Maryam Tamaddon^1^, Gordon Blunn^2^, Rongwei Tan^3^, Pan Yang^3^, Xiaodan Sun^4^ , Shen-Mao Chen^1^, Jiajun Luo^1^, Ziyu Liu^1^, Ling Wang^5^, Dichen Li^5^, Ricardo Donate^6^, Mario Monzón^6^, Chaozong Liu^1*^

^1^ Institute of Orthopaedic and Musculoskeletal Science, Royal National Orthopaedic Hospital, University College London, Stanmore HA7 4LP, UK

^2^ School of Pharmacy and Biomedical Sciences, University of Portsmouth, Portsmouth PO1 2DT, UK

^3^ Guangdong Engineering Research Center of Implantable Medical Polymer, Shenzhen Lando Biomaterials Co., Ltd., Shenzhen 518107, China

^4^ School of Materials Science and Engineering, Tsinghua University, Beijing 100084, China

^5^ State Key Laboratory for Manufacturing System Engineering, School of Mechanical Engineering, Xi’an Jiaotong University, Xi’an 710054, China

^6^ Departamento de Ingeniería Mecánica, Grupo de Investigación en Fabricación Integrada y Avanzada, Universidad de Las Palmas de Gran Canaria, Campus Universitario de Tafira s/n, 35017 Las Palmas, Spain

**Corresponding author**: Chaozong Liu: [Chaozong.Liu@ucl.ac.uk](mailto:Chaozong.Liu@ucl.ac.uk)

**Online Resource 1**

Comparison of the cellular activity on two different PLA scaffold architecture: 8 layers with filaments placed at 45 degrees and 90 degrees.

**Experimental procedure**

The PLA scaffolds were 3D printed, treated and sterilised as described previously in the main text.

500,000 sheep bone marrow mesenchymal stem cells (passage 5) were used for each scaffold. 250,000 cells were suspended in 50ul of cell culture media and placed on top of scaffolds. The scaffolds were incubated for 1hr to allow adhesion, after which they were flipped so that another 250,000 of cells in 50ul of media were seeded on the other side of scaffolds. The scaffolds were then incubated at 37 ᵒC, 5% CO_2_, and processed for imaging/analysis at 1, 3, 7, 21 and 28 days as described in the main text. Cells at 21 days were imaged with Scanning Electron Microscopy after fixation with 4% PFA for 20 min, dehydration through a series of alcohol (30% to 100%), and HDMS. The samples were mounted on to SEM stubs, coated with gold-palladium, and observed under SEM with acceleration voltage of 5kV.

All experimental groups had a sample size of n=3 and statistical significance was determined by performing two-way ANOVA with Sidak post-hoc test, with a confidence level of 95% (significance accepted at a level of ρ < 0.05).

**Results and discussion**

Live/Dead staining of cells on two types of 3D printed PLA scaffolds showed cell viability on both designs up to 28 days. After an initial difference in the cell attachment between two designs, the sample with filaments at 90 degrees showed similar or higher cell proliferation over 28 days. SEM micrographs show that the cells have attached to PLA filaments in both cases, where they proliferated over time and occupied most of the surfaces. They seemed to be aligned to the long direction of filaments and particularly attach to the corners to bridge them. Overall, a higher cell proliferation was observed and maintained on the samples with filaments at 90 degrees and this architecture was selected for the final multi-material scaffold.


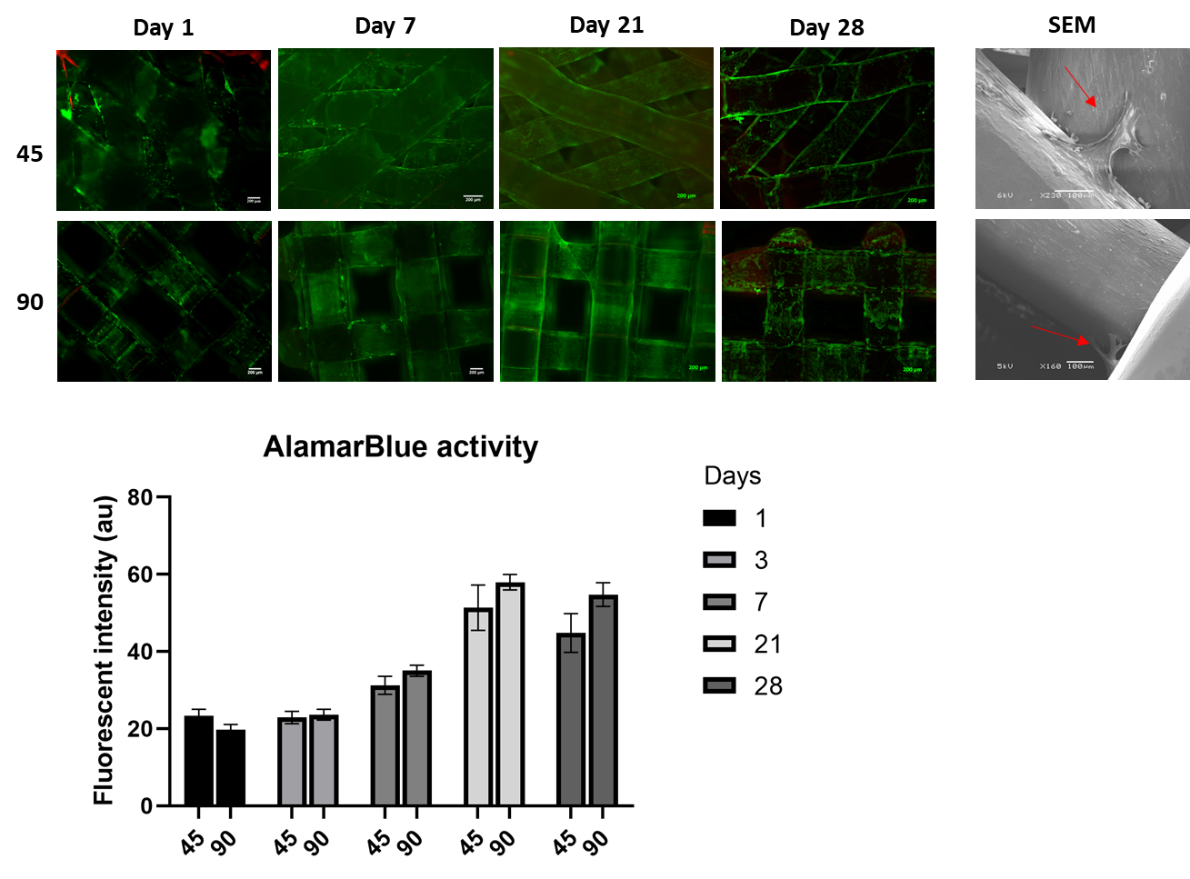


Figure 1 Cell behaviour on two different designs of PLA. Live/Dead staining shows cell viability on both types throughout 21 days. SEM shows cell attachment and bridging between layers (red arrow). AlamarBlue shows higher cell metabolic activity on design with 90-degree filaments.

**Online Resource 2**

Scoring system for macroscopic appearance of regenerated cartilage

| Criteria | Description | Score |
| --- | --- | --- |
| Tissue colour | Similar to native cartilage, white (minimum red discoloration) | 8-10 |
|  | Intermediate, some discoloration, less than 50% white | 5-7 |
|  | Complete discoloration | 0-4 |
| Homogeneity | Homogenous in all areas or in>80% areas | 8-10 |
|  | Homogenous in 50-80% of areas | 5-7 |
|  | Not homogenous or less than 50% | 0-4 |
| Smoothness | Smooth surface, or some small fissures/fibrillation | 8-10 |
|  | Fibrillated surface | 5-7 |
|  | Rough surface with deep/large fissures | 0-4 |
| Edge integration (new tissue relative to native cartilage) | Complete integration or slight demarcating border <20% of the circumference | 8-10 |
|  | Demarcation between 20-50% | 5-7 |
|  | Demarcation more that 50% of the circumference | 0-4 |
| Fill | Complete fill, or more that 70% of the defect area | 8-10 |
|  | Fill between 50-70%, slight depression | 5-7 |
|  | Fill less than 50%, complete depression or overgrowth | 0-4 |

**Online Resource 3**

Modified ICRSII score

|  | Histological parameters | Score |
| --- | --- | --- |
| 2 | Matrix staining (metachromasia) | 0%: No staining  100%: Full metachromasia |
| 3 | Cell morphology | 0%: No round/oval cells  100%: Mostlt round/oval cells |
| 4 | Chondrocyte clustering (4 or more grouped cells) | 0%: Present  100%: Absent |
| 5 | Surface architecture | 0%: Delamination or major irregularity  100%: Smooth surface |
| 11 | Vascularization (within the repaired tissue) | 0%: Present  100%: Absent |
| 12 | Surface/superficial assessment | 0%: Total loss or complete disruption  100%: Resembles intact articular cartilage |
| 13 | Mid/deep zone assessment | 0%: Fibrous tissue  100%: Normal hyaline cartilage |
| 14 | Overall assessment | 0%: Bad (fibrous tissue)  100%: Good (hyaline cartilage) |

**Online Resource 4**

The primer sequences of the targeted genes and the internal control gene

| Gene | F | R |
| --- | --- | --- |
| β2 microglobulin | CCAGAAGATGGAAAGCCAAA | AGCGTGGGACAGAAGGTAG |
| COL2a | CCTCAAGAAGGCTCTGCTCA | ATGTCAATGATGGGGAGACG |
| ACAN | TAGGTGGCGAGGAAGACATC | AAACGTGAAAGGCTCCTCAG |
